# Do female dieters have an “eating disorder” self-schema?

**DOI:** 10.1186/s40337-016-0103-5

**Published:** 2016-05-16

**Authors:** Sarah Greer, Myra Cooper

**Affiliations:** Isis Education Centre, University of Oxford, Warneford Hospital, Oxford, OX3 7JX UK; Richmond Hospital, 7000 Westminster Hwy, Richmond, BC V6X 1A2 Canada

**Keywords:** Eating disorders, Dieting, schema, self-schema, Core beliefs, Information processing

## Abstract

**Background:**

The processing of schema-related information is important in the maintenance of specific eating disorder (ED)-related belief systems and psychopathology. To date, most research on differences in the processing of ED schematic information has used interview or self-report questionnaire measures. Dieting is a known risk factor for EDs and dieters have been included in some studies. However, they have not been compared with non-dieters on a novel, objective measure of ED related schema processing.

**Methods:**

The current study recruited healthy female volunteers from the community and divided them into dieting (*n* = 25) and non-dieting (*n* = 24) groups using rigorous criteria. ED self-schemas with content unrelated to eating, weight and shape were measured using a self-schema processing task.

**Results:**

Dieters endorsed significantly more ED relevant words compared to non-dieters, whereas non-dieters rejected significantly more ED relevant words compared to dieters. Reaction times to endorsements and rejections were non-significant when the two groups were compared. In a surprise recall task, dieters recalled significantly more ED relevant words.

**Conclusion:**

The results of this study support the presence of ED self-schemas with negative content unrelated to eating, weight and shape in otherwise healthy dieters. Implications for future research and the early identification of individuals vulnerable to EDs are discussed.

## Background

Self-schemata are “cognitive generalisations about the self, derived from past experience that organise and guide the processing of self-related information contained in the individual’s social experiences” ([1], p64). Identifying and modifying maladaptive self-schemata (also termed core or negative self-beliefs), is now often considered to be a key focus of eating disorder (ED) treatment [2–4]. As described by Young [5, 6] these schemata typically develop early in life, and although commonly associated with personality disorders, also have a strong association with EDs (for example, as measured by the Young Schema Questionnaire (YSQ, [7]) [8]. Although it has proved difficult to identify schema patterns typical of eating or other psychiatric disorders using the YSQ, self-schemata or negative self-beliefs that may be uniquely associated with EDs, compared to depressive and anxious patients and symptoms have been found using exploratory, data driven work [9, 10]. This is important as people with EDs often have high levels of anxiety and depression, and it is theoretically and clinically useful to identify core beliefs particularly characteristic of the ED. In terms of measurement, with the exception of Benas and Gibb [11], who used computer-based tasks to measure reaction times to specific content, most studies have assessed self-schema in EDs using self-report questionnaires or semi-structured interviews (e.g., [10, 12, 13]). Experimental designs, for example, Markus’s [1] “Me/Not” paradigm, have not generally been used but may provide a more objective assessment of any biased self-processing relevant to EDs.

Dieting is a risk factor for EDs, and a strong predictor of ED symptoms [14, 15]. Dieters may be best defined as those *attempting* to restrict food intake rather than only those succeeding; as attempt rather than success is important in cognitive behaviour models of EDs [16]. Dieting measured using the Eating Disorder Examination Questionnaire (EDE-Q; [17]) predicted the development of an ED [16]; thus the EDE-Q may be a particularly useful measure of dieting in EDs. Given the role of dieting in EDs, one group who may have accessible ED related self-schemata are dieters.

The notion dieters have an active ED self-schema was tested by Pringle, Harmer and Cooper [18]. They used a self-schema processing task (SSPT) based on Markus’s [1] “Me/Not Me” paradigm. The task measured endorsement and reaction times to ED relevant, depression relevant, generic negative and generic positive/neutral words, drawn from Cooper and Cowen’s [9] work on self-schema. An ED-relevant schema was associated with higher levels of ED-related symptoms [18]. As described by Cooper and Cowen, an ED-relevant self schema contains extreme negative content, that is unrelated to eating, weight and shape, and is at least partly distinct from the negative self schema associated with depression [9]. A follow-up study indicated that an ED self-schema was related to risk of developing an ED twelve months later [19]. An ED self-schema may thus represent one mechanism through which dieting increases vulnerability to developing an ED.

The study by Pringle and colleagues [18] used a cross sectional design. To date no study has investigated whether dieters have an ED self-schema, compared to non-dieters. In the current study it was hypothesised that dieters will endorse more ED self-schema-relevant words and show faster reaction times for “Me” endorsements to ED words compared to depression, generic negative words and generic positive/neutral words, than non-dieters. It was also hypothesised that dieters would have higher levels of recall of words related to ED self-schema compared to all three other groups. Dieter and non-dieter groups were rigorously defined to ensure they did not contain participants with EDs or other psychiatric disorders; thus an ‘at risk’ rather than symptomatic group enabled potential vulnerability to be tested.

## Method

### Recruitment

Following Oxford University ethical approval, women aged 18 to 35 were recruited through advertisements that sought healthy volunteers. Advertisements were placed in online newspapers, student newsletters, social networking sites, and notice boards at colleges and gyms. Presentations were made at undergraduate lectures at local universities, gym classes and meetings of dieting organisations (e.g., “Weight Watchers”). Advertisements sought women who were actively dieting, attempting to diet or trying to eat healthily as well as those who were not (i.e., non-dieters). Potential participants responded by completing an online survey, which was used to screen to ensure basic study criteria were met.

### Screening

Inclusion criteria were: Female, between and including the ages of 18 – 35 years.

Exclusion criteria were: Psychiatric history, including an ED; self-declared proficiency in English less than comfortably fluent.

### Participants

**Fig. 1:**
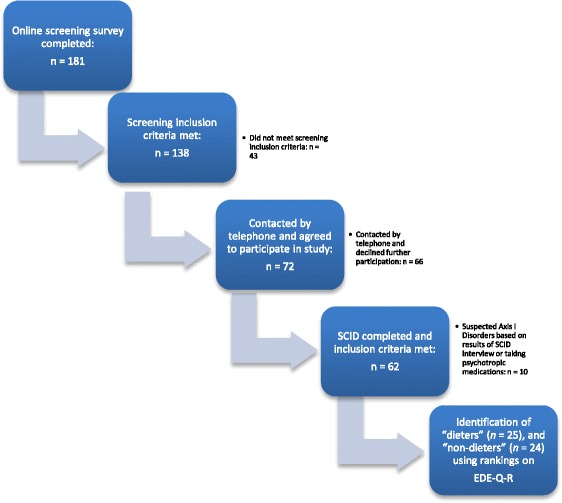
Flow chart showing recruitment pathway and selection of participants

Of the 181 women who began the online survey, 138 met screening criteria, completed the survey, and indicated willingness to be contacted for further testing. Of these, 72 agreed to meet and completed the Structured Clinical Interview for DSM-IV Axis I disorders (SCID; [20]). Ten met Axis I criteria, or were taking medication that might affect task performance [21]. These people were excluded, leaving 62 potential participants. All participants gave informed consent at each stage of the study. Figure 1 depicts the screening and participant selection process.

### Creating dieting and non-dieting groups

Technical difficulties were experienced with one participant’s data. Another participant’s data was removed after she disclosed difficulties understanding the SSPT words. The sample then included 60 females. A ranking was established using scores on the Restraint subscale to identify participants with the highest and lowest dieting behaviours. Two groups were thus identified for the final analyses: “dieters” (*n* = 25), and “non-dieters” (*n* = 24); numbers were in line with previous research in this area and adequate for the analyses planned [22]. Dieters had a Restraint subscore of 2.4 and above and non-dieters had a score of 1.4 and below.

### Power

An exact calculation of number of participants needed and power could not be made due to a lack of previously relevant studies. However, Cohen’s (1988) rule of thumb suggested 26 participants in each cell for 80 % power using analysis of variance, assuming a large effect size, and with alpha set at 0.05.

### Measures

The Self-Schema Processing Task (SSPT) [18]. The SSPT task involves four sets of 30 “self words” matched for length and word frequency: ED relevant (e.g., evil, repulsive), depression relevant (e.g., numb, excluded), generic negative (e.g., hostile, bossy) and generic positive/neutral (e.g., honest, pleasant). The ED and depression relevant words were taken from Cooper and Cowen [9], and negative and positive words from Anderson [23]. The groups of words were matched for number of letter in each word and word frequency using the MRC Psycholinguistic database [18].

Participants were shown a word on a laptop screen and asked to indicate whether they felt the word described or applied to them, i.e., was “me” or “not me”, by pressing labelled keys on the keyboard. A “fixation cross” appeared on the screen for 1 s and disappeared to reveal the target word [24]. Words were presented for 500 milliseconds, with participants asked to respond as quickly and as accurately as possible. Two scores were used here, reaction time in milliseconds and number of words endorsed (and subsequently, recalled).

Eating Disorder Examination-Questionnaire-Restraint Subscale (EDE-Q-R). The EDE-Q is a 36 item self-report questionnaire derived from the Eating Disorder Examination (EDE) interview [25]. It has good reliability and acceptable criterion validity, including in women in community samples [26]. A measure of dieting behaviour was obtained here using the “Restraint” subscale.

Eating Attitudes Test (EAT-26) [27]. This is a widely used self-report measure of ED symptoms. High internal consistency (alpha = .90) and acceptable criterion-related validity have been reported [28].

Hospital Anxiety and Depression Scale (HADS). The HADS [29] is a self-assessment scale of anxious and depressive symptoms with well-established psychometric properties, and detects depression and anxiety in individuals in a variety of settings [30].

Rosenberg Self Esteem Scale (RSE). The RSE is a widely used self-report measure of global self-esteem. It is composed of 10 statements related to self-worth and self-acceptance [31]. It has high reliability and validity and has been called, “the standard by which new self-esteem measures are evaluated” ([32], p.123).

National Adult Reading Test (NART; [33]). This is a widely used estimate of general intelligence or IQ, with good psychometric properties. It was included because of potential confounds with the complexity of the words used in the SSPT and with level of education and dieting behaviour.

Visual Analogue Scales (VAS). These measured emotions on the day of testing. The emotions measured were: happy, sad, angry, frightened, anxious, disgusted and surprised. These were included because of potential confounds between mood at time of testing and SSPT responses.

### Procedure

Testing (individually based) began with administering the SSPT, followed by “filler” tasks to provide a 5–10 min delay before the surprise free recall task. The session also included the NART; 32), and a measure of emotions using the VAS.

### Data analysis

Mixed design ANOVAs were planned for endorsements, reaction time and recall on the SSPT (group × “Me/Not Me” × word category), provided data met the relevant assumptions.

## Results

### Participant characteristics

Of the final sample (*n* = 49), most were White/Caucasian (90 %), followed by Mixed (6 %), Asian or British Asian (2 %) and Other (2 %). Information on participants’ age, Body Mass Index (BMI), EAT-26 scores, and NART scores is provided in Table 1. BMI data was missing for one participant in the dieting group. Mean EAT-26 scores for both dieting and non-dieting groups were in the normal range, i.e., below the clinical cut-off of 20 points [27], supporting the non-clinical definition of these groups.

**Table 1:** Demographic and between-group statistics for the dieting and non-dieting groups

	Dieters	Non-dieters		
	(*n* = 25)	(*n* = 24)		
	Mean (SD)	Mean (SD)	*t* value	Significance level
Age (years)	23.48 (4.8)	25.67 (3.5)	1.81	.08
Current BMI	22.85 (2.5)	24.28 (4.8)	1.29	.20
EDE-Q-R scores	3.57 (1.0)	0.77 (0.5)	12.82	.0001^***^
EAT-26 scores	14.72 (13.8)	11.42 (9.9)	.97	.34
Anxiety (HADS)	5.12 (3.5)	5.21 (3.2)	.09	.93
Depression (HADS)	2.36 (2.3)	2.38 (2.2)	.02	.98
Estimated IQ (NART)	108.44 (8.2)	105.7 (5.6)	1.38	.18
Self-Esteem (RSE)^a^	17.96 (6.6)	20.17 (4.6)	1.36	.18

*BMI* body mass index, *EAT-26* eating attitudes test, *EDE-Q-R* eating disorders examination questionnaire (Restraint subscale), *HADS* hospital anxiety and depression scale, *IQ* intelligence quotient, *NART* National Adult Reading Test, *RSE* (rosenberg self-esteem scale), *SD* standard deviation

^a^Higher scores indicate higher levels of self-esteem

^***^ *p* < .0001

Also important for a healthy, non-clinical sample, mean scores for anxiety and depression symptoms on the HADS were in the normal range (below cut-off of 7 points) [29]. No other variables differed significantly between the dieters and non-dieters. This included years in education (*X*^2^ = 1.25, p = .52) and English as a mother tongue (*p* = .66),

#### EDE-Q Restraint scores

These can be seen in Table 1. As expected, the dieting group had a significantly higher score on the EDE-Q-R than the non-dieting group (*t* (1, 47) = −12.82, *p* < .001).

### SSPT: endorsements

Endorsement data can be seen in Table 2.

**Table 2:** Mean number of words endorsed as “Me” and “Not me” on the SSPT

	Dieters		Non-dieters	
	(*n* = 25)		(*n* = 24)	
	“Me”	“Not Me”	“Me”	“Not Me”
	Mean (SD)	Mean (SD)	Mean (SD)	Mean (SD)
Eating disorder relevant	2.4 (2.8)	27.6 (2.8)	0.5 (.72)	29.5 (0.7)
Depression relevant	3.5 (5.7)	26.4 (5.9)	1.46 (2.0)	28.5 (2.0)
Generic negative	4.8 (4.4)	25.1 (4.4)	3.7 (2.8)	26.21 (3.0)
Generic positive/neutral	22.8 (5.0)	7.0 (4.8)	24.2 (3.8)	5.8 (3.8)

*SD* standard deviation, *SSPT* self-schema processing task

Assumptions of normality were not met and there was a significant amount of variance between groups. This prevented meaningful interpretation of a mixed design ANOVA (group x “Me”/”Not Me” endorsements x word category). Separate ANOVAS^1^ were therefore conducted for “Me”/”Not Me” endorsements in each of the four categories of words (i.e., ED relevant, depression relevant, generic negative, and generic positive/neutral), with a between-group factor of dieting/non-dieting. There was a significant group effect for number of ED relevant words endorsed as “Me”, (*F* (1, 47) = 10.71, *p* = .002). No other significant group effects were found. All statistics can be seen in Table 3.

**Table 3:** F-Scores for group effects on endorsements of word categories and reaction times (SSPT)

	Endorsements				Reaction Times			
	“Me”	*P*	“Not Me”	*P*	“Me”	*P*	“Not Me”	*P*
ED relevant	10.71	.002**	10.25	.002**	1.19	.28	2.21	.14
Depression relevant	2.94	.09	2.89	.09	.44	.51	.98	.33
Generic negative	1.14	.29	1.62	.21	.69	.41	1.99	.17
Generic positive/neutral	1.17	.28	1.02	.32	1.00	.32	1.10	.30

*ED* eating disorder, *SSPT* self-schema processing task

**p < .01

Post hoc tests indicated that dieters endorsed significantly more ED-relevant words as “Me”, than non-dieters (*U* = 156.0, *z* = −3.05, *p = .002*, *r* = −.44). No other comparisons were significant.

### SSPT: reaction times

A mixed design (group x “Me”/”Not Me” reaction times x word category) ANOVA was performed. However, owing to the forced-choice design (i.e., “Me” or “Not Me”) of the SSPT, participants could potentially respond identically to all words within the same category. Unfortunately, this led to a large amount of missing data as a number of participants responded “Not Me” to all words in some categories. This meant that nearly half (*n* = 23) the sample could not be included, resulting in an underpowered analysis (*n* = 26) that could not be meaningfully interpreted.

In an exploratory analysis, the mean reaction times for categories of words and dieting groups were analysed using ANOVAs to look at between group differences, as for endorsements (see data in Table 4). Dieters and non-dieters’ mean reaction times to words from all four categories were not significantly different (all *p* values > .1).

**Table 4:** Mean reaction times in milliseconds on the SSPT

	Dieters		Non-dieters	
	(*n* = 14)		(*n* = 12)	
	“Me”	“Not Me”	“Me”	“Not Me”
	Mean (SD)	Mean (SD)	Mean (SD)	Mean (SD)
Eating disorder relevant	703.6 (546.9)	414.2 (193.9)	536.7 (525.1)	338.5 (160.3)
Depression relevant	602.7 (648.7)	511.6 (375.6)	487.2 (565.9)	422.9 (230.7)
Generic negative	663.6 (375.5)	482.6 (243.5)	573.3 (385.5)	394.6 (187.9)
Generic positive/neutral	517.6 (241.7)	778.3 (415.9)	448.5 (242.3)	665.0 (333.2)

*SD* standard deviation, *SSPT* self-schema processing task

### Recall task

**Fig. 2:**
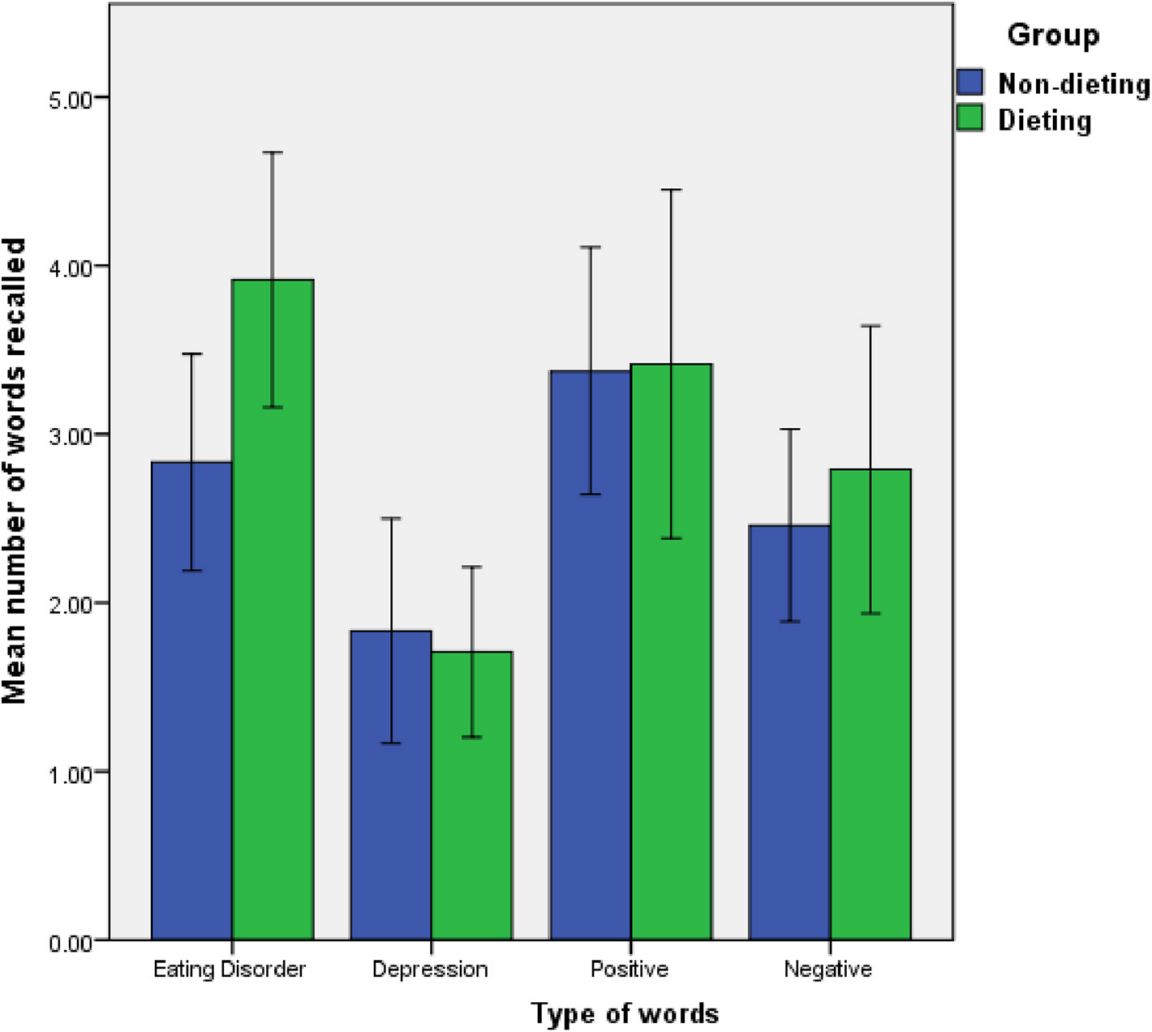
Mean number of words of each type recalled by non-dieters and dieters

A repeated measures ANOVA indicated that the pattern of dieters’ recall of words from each of the four categories did not differ significantly from that of non-dieters’ (*F* (3, 141) = 1.344, *p =* .26), and these are represented in Fig. 2.

Mean number of words recalled by participants in the SSPT surprise recall task can be seen in Table 5. Post hoc tests indicated that recall of ED relevant, but no other, words differed significantly between groups (*t* (1, 47) = −2.31), *p* = .025), with dieters recalling more ED relevant words than non-dieters.

**Table 5:** Mean number of words recalled by participants from the SSPT

	Dieters	Non-dieters
	(*n* = 25)	(*n* = 24)
	Mean (SD)	Mean (SD)
Eating disorder relevant*	3.92 (1.8)	2.83 (1.5)
Depression relevant	1.80 (1.3)	1.83 (1.6)
Generic negative	2.68 (2.1)	2.46 (1.4)
Generic positive/neutral	3.40 (2.4)	3.38 (1.7)

*SD* standard deviation

Difference between groups, *p < .05

### VAS emotions

Of the VAS emotions only “sad” scores differed significantly, *U* = 192.50, *z* = −2.15, *p* = .031, *r* = −.31. Dieters reported greater feelings of sadness on the day of testing than non-dieters.

### Covariates

Owing to the significant difference between dieting and non-dieting groups on the “sad” emotion VAS, this variable was included in the significant analyses as a covariate to determine its potential impact. “Sad” scores were associated with endorsements of ED relevant words (*F* (1, 46) = 16.71, *p* < .001); however, the main between-group effect of ED relevant words on endorsements remained significant (*F* (1, 46) = 5.06, *p* = .03) with “sad” as a covariate.

## Discussion

As expected, the dieting group differed significantly from the non-dieting in greater endorsement of ED relevant words, but not depression relevant, generic negative and generic positive/neutral words, on the SSPT. This finding adds to that of Pringle et al. [18], who found that longer reaction times to reject ED relevant words as “Not Me” were predictive of sub-clinical ED symptoms in dieters. The current findings indicate that dieters with no significant ED symptoms also endorse significantly more ED relevant words as “Me” when compared to non-dieters.

Unlike Pringle’s study [18] there were no significant differences between groups on reaction times measures, for either endorsements or rejections of ED relevant words. However, this data included missing values for several participants. These analyses were therefore most likely underpowered. Imputing missing data was considered but thought inadvisable as the data imputation method that seemed most appropriate is normally used in repeated measures designs [34], and this was not the method used here. To improve power, future studies might consider using pre-designated and equally occurring categories (e.g., identifying the colour of a word). However, using a method without “Me” and “Not Me” endorsements/rejections might be seen as differing significantly from the original theory underlying the schema paradigm [1]. More radically, it could be asked whether both reaction times and endorsements are always necessary to identify the presence of a schema, or even whether endorsements are more valid (and easier to measure) demonstrations of schematic functioning.

While ratings were relatively low, dieters scored significantly higher on a VAS measure of sadness than non-dieters. The mean difference in “sad” ratings between groups was significantly associated with number of endorsements of ED relevant words. Nevertheless, the main effect of endorsement and dieting group remained significant after controlling for sadness, suggesting that sadness did not provide a complete explanation for the difference between the groups on endorsements. There was no significant relationship between “sad” ratings and the number of ED words recalled by dieters or non-dieters. Greater recall by the dieters could not therefore be attributed to any differences between the two groups in VAS sad ratings.

Given the high comorbidity of EDs with mood and anxiety disorders [35], higher “sadness” ratings are not wholly unexpected. However, it is also possible that this emotion is linked to the experience of dieting, as dieters are thought to hold a negative view of the self [36]. Theoretical models of EDs postulate an association between core belief activation and distress [3], providing some support for this assertion in an “at risk” group.

One possibility is that these “unrelated content” tasks might be behavioural biomarkers of, for example, risk for EDs (as suggested by the data in Pringle et al., 18). More generally, these data add to the research suggesting an important role for ED-relevant self-schemas, in both ED groups and as a dimensional construct [9, 12, 18, 19]. Future research in dieters and other groups “at risk” of an ED might usefully include measures investigating content not only related to eating, weight and shape, but also to generic or ED-related self-schemas.

The study had some limitations. The sample size was relatively small, and this was particularly true for investigating self-schema reaction times. Nevertheless, schema processing differences were found between the two groups on number of endorsements and in the surprise recall test, thus two out of the three main hypotheses were confirmed, with only differences in reaction time between the dieters and non-dieters not proving significant. A measure of self-reported hunger was not administered on the day of testing given dieters who do not demonstrate ED symptoms are not suffering from the effects of semi-starvation [37]. Nevertheless, more subtle differences in hunger may have been present and future studies might usefully measure this. Self-report data were used to calculate BMI; ideally height and weight should have been measured on the day of testing.

The planned data analytic strategy was altered as data on the SSPT did not meet the assumptions for parametric analyses. While it may subsequently have been desirable to adjust significance levels for the increased number of analyses conducted, the study was an exploratory one and, as such, no adjustments were made in order to minimise the risk of error arising from this procedure [36]. Post hoc comparisons were conducted in the absence of a significant omnibus effect; again, while this can be inadvisable, it can also be a useful analysis to minimise the risk of a Type 1 error, which it is important to guard against in an exploratory study such as this [38]. Finally, the nature of the data did not allow analysis of ‘me’ and ‘not me’ data together, but inspection of the means indicates that dieters and non-dieters both appeared to endorse many more negative words as ‘not me’, than ‘me’. This is perhaps not surprising in a relatively healthy population.

The study had several strengths. Participants were rigorously screened and had higher dietary restraint scores than some previous studies of dieters [16, 17]. Screening ensured that they did not have a psychiatric disorder, including an ED. Findings of interest, therefore, can be attributed to the “at risk” status rather than the presence of any significant ED symptoms.

## Conclusion

Future research may benefit from investigating self and ED schema processing in those with EDs. A depressed group might be a useful comparison in any such study. It may be possible to establish some criteria by which schematics and aschematics in those “at risk,” might be identified. Given that not all dieters will develop an ED, investigating the profile of each (schematic or aschematic) might refine our notion of who is most at risk [18], and could benefit clinical outcomes through the early identification of vulnerable individuals [37].

## Footnotes

1 This is considered equivalent to an independent sample *t*-test of between-group means.
